# The impact of mindfulness training in early adolescence on affective executive control, and on later mental health during the COVID-19 pandemic: a randomised controlled trial

**DOI:** 10.1136/ebmental-2022-300460

**Published:** 2022-07-07

**Authors:** Darren Dunning, S Ahmed, L Foulkes, C Griffin, K Griffiths, J T Leung, J Parker, Blanca Piera Pi-Sunyer, A Sakhardande, M Bennett, C Haag, Jesus Montero-Marin, D Packman, Maris Vainre, P Watson, The MYRIAD Team, Willem Kuyken, J Mark G Williams, Obioha C Ukoumunne, Sarah-Jayne Blakemore, Tim Dalgleish

**Affiliations:** 1 Medical Research Council Cognition and Brain Sciences Unit, Cambridge University, Cambridge, Cambridgeshire, UK; 2 Institute of Cognitive Neuroscience, University College London, London, UK; 3 Teaching, Research and Innovation Unit, Parc Sanitari Sant Joan de Déu, Sant Boi de Llobregat, Spain; 4 Department of Psychiatry, University of Oxford, Oxford, Oxfordshire, UK; 5 College of Medicine and Health, University of Exeter, Exeter, UK; 6 NIHR CLAHRC South West Peninsula, University of Exeter Medical School, Exeter, UK; 7 Department of Psychology, University of Cambridge, Cambridge, UK

**Keywords:** child & adolescent psychiatry

## Abstract

**Background:**

Previous research suggests that mindfulness training (MT) appears effective at improving mental health in young people. MT is proposed to work through improving executive control in affectively laden contexts. However, it is unclear whether MT improves such control in young people. MT appears to mitigate mental health difficulties during periods of stress, but any mitigating effects against COVID-related difficulties remain unexamined.

**Objective:**

To evaluate whether MT (intervention) versus psychoeducation (Psy-Ed; control), implemented in after-school classes: (1) Improves affective executive control; and/or (2) Mitigates negative mental health impacts from the COVID-19 pandemic.

**Methods:**

A parallel randomised controlled trial (RCT) was conducted (Registration: https://osf.io/d6y9q/; Funding: Wellcome (WT104908/Z/14/Z, WT107496/Z/15/Z)). 460 students aged 11-16 years were recruited and randomised 1:1 to either MT (N=235) or Psy-Ed (N=225) and assessed preintervention and postintervention on experimental tasks and self-report inventories of affective executive control. The RCT was then extended to evaluate protective functions of MT on mental health assessed after the first UK COVID-19 lockdown.

**Findings:**

Results provided no evidence that the version of MT used here improved affective executive control after training or mitigated negative consequences on mental health of the COVID-19 pandemic relative to Psy-Ed. No adverse events were reported.

**Conclusions:**

There is no evidence that MT improves affective control or downstream mental health of young people during stressful periods.

**Clinical implications:**

We need to identify interventions that can enhance affective control and thereby young people’s mental health.

**What is already known on this topic**

- A key mechanism of action of mindfulness-based interventions in delivering mental health benefits is hypothesised to be enhancing executive control in affectively or socially charged contexts.
- Experimental research to date has tended to focus on executive control as a cold cognitive capacity rather than on its selective application in socially or affectively charged contexts.

**What this study adds**

- Using an adequately powered RCT with experienced teachers and good engagement from volunteer student participants we found no support for the hypothesis that a widely used mindfulness-based intervention for young people (.b) compared with an active psychoeducation control condition (Psy-Ed), would deliver superior changes in affective executive control.
- An opportunistic extension of the trial into the period of the COVID-19 pandemic found no support for .b offering superior protection against worsening mental health relative to Psy-Ed.

**How this study might affect research, practice or policy**

- The next generation of research needs to consider what works, for whom, in addition to how, as well as considering key contextual and implementation factors.

## Introduction

Mindfulness training (MT) involves systematic practice in focusing attention in a sustained and intentional way.^1^ MT has been shown to prevent and/or improve mental health problems in clinical and non-clinical adult samples, and MT in young people appears promising as a preventive intervention.^2 3^ However, less is known about the mechanisms that drive these MT-related improvements in mental health.^4^ Understanding the mechanisms of MT is essential as it allows further refinement of MT to target those mechanisms, and can inform other interventions that also target the same mechanisms.

A compelling proposal is that positive effects of MT on mental health are mediated by improvements in executive control in affective contexts; specifically, improvements in the ability to allocate mental resources in the service of pertinent goals in the face of affectively salient distraction.^4 5^ Such affective executive control is typically measured using experimental cognitive tasks that assay executive control separately in affectively laden and neutral contexts. The degree to which affective, relative to neutral, contexts lead to a performance decrement is the index of affective executive control. Importantly, affective executive control appears to improve following systematic training^6 7^ and to be compromised in individuals suffering from mental health difficulties, including in younger samples.^8^

Although several studies have used experimental tasks to measure changes in diverse executive skills following MT, they tend to have used emotionally neutral tasks that lack the requisite affective condition for indexing performance in affective contexts. Results are mixed with studies reporting either gains or no effects following MT in skills including sustained attention, selective attention/inhibition, and working memory.^3^

## Objective

Here, we report a randomised controlled trial (RCT) to evaluate whether MT improves performance on experimental and self-report measures of affective executive control in young people aged 11–16 years. The RCT compared MT to a psychoeducation control condition (Psy-Ed), comparable in length and complexity to MT. Importantly, a Psy-Ed control (Student Success Skills (SSS)^9^) was chosen that should deliver comparable postintervention effects to MT on mental health outcomes.^9^ This allows us to disentangle any differential effects of MT versus Psy-Ed on affective executive control from effects on mental health.

Our hypothesis was that MT, relative to Psy-Ed, would deliver greater improvements in affective executive control. If this hypothesis was supported, it would allow us to test a follow-on interactive mediation hypothesis^10^ that, in contrast to the Psy-Ed arm, any changes in mental health outcomes in the MT trial arm would be differentially driven by improvements in this key mechanism of affective executive control.

Following completion of the RCT, the onset of the COVID-19 global pandemic led to significant social changes in the UK for young people, including a national lockdown involving school closures. We know from trials in older students that the key skills taught during MT can protect against deterioration in mental health during periods of stress, even if there are no apparent MT-related mental health benefits prior to the onset of stress.^11^ Due to the incumbent stress and potential negative impacts on mental health of the COVID-related pandemic,^12^ we decided to extend the RCT to include an additional wave of data collection following the first UK COVID-19 lockdown in summer 2020. We hypothesised that MT, relative to Psy-Ed, delivered prior to the pandemic, would mitigate the subsequent development of pandemic-related mental health difficulties.

## Methods

The trial is reported in accordance with CONSORT guidelines. The full study design and procedures, including the COVID-related extension, are presented in the trial protocol.^13^

### Study design and participants

A superiority randomised controlled, parallel-group mechanisms trial—as part of the broader My Resilience in Adolescence (MYRIAD) programme of work focused on evaluating and understanding MT in adolescence—compared MT (intervention) with Psy-Ed (control) to test the hypothesis that MT would have a superior effect on measures of affective executive control at postintervention. Following the onset of the COVID-19 pandemic, we extended the trial to include an additional mid-pandemic assessment to test the hypothesis that MT is superior to Psy-Ed in mitigating symptoms of mental ill health and promoting well-being following the onset of the pandemic.

There were three key assessment points: Time 1 (T1, January 2017 to March 2019): prerandomisation baseline—a face-to-face assessment 1–2 weeks prior to the start of the interventions; Time 2 (T2, March 2017 to May 2019): Postintervention—a face-to-face assessment and the primary end point for affective executive control outcomes; Time 4 (T4, June–July 2020): 20–44 months after the interventions finished, following the first UK lockdown during the COVID-19 pandemic—an online assessment and the primary end point for the mental health outcomes. The reason for the large time window for this T4 assessment was that all participants were assessed at the same time during July 2020, whereas the original trial was spread over a 26-month recruitment period. An additional online assessment of mental health outcomes (Time 3, T3: June 2017 to August 2019) was completed 3 months after the interventions to enable putative analyses of longer-term mediating effects of changes in affective executive control on changes in mental health.

**Figure 1:**
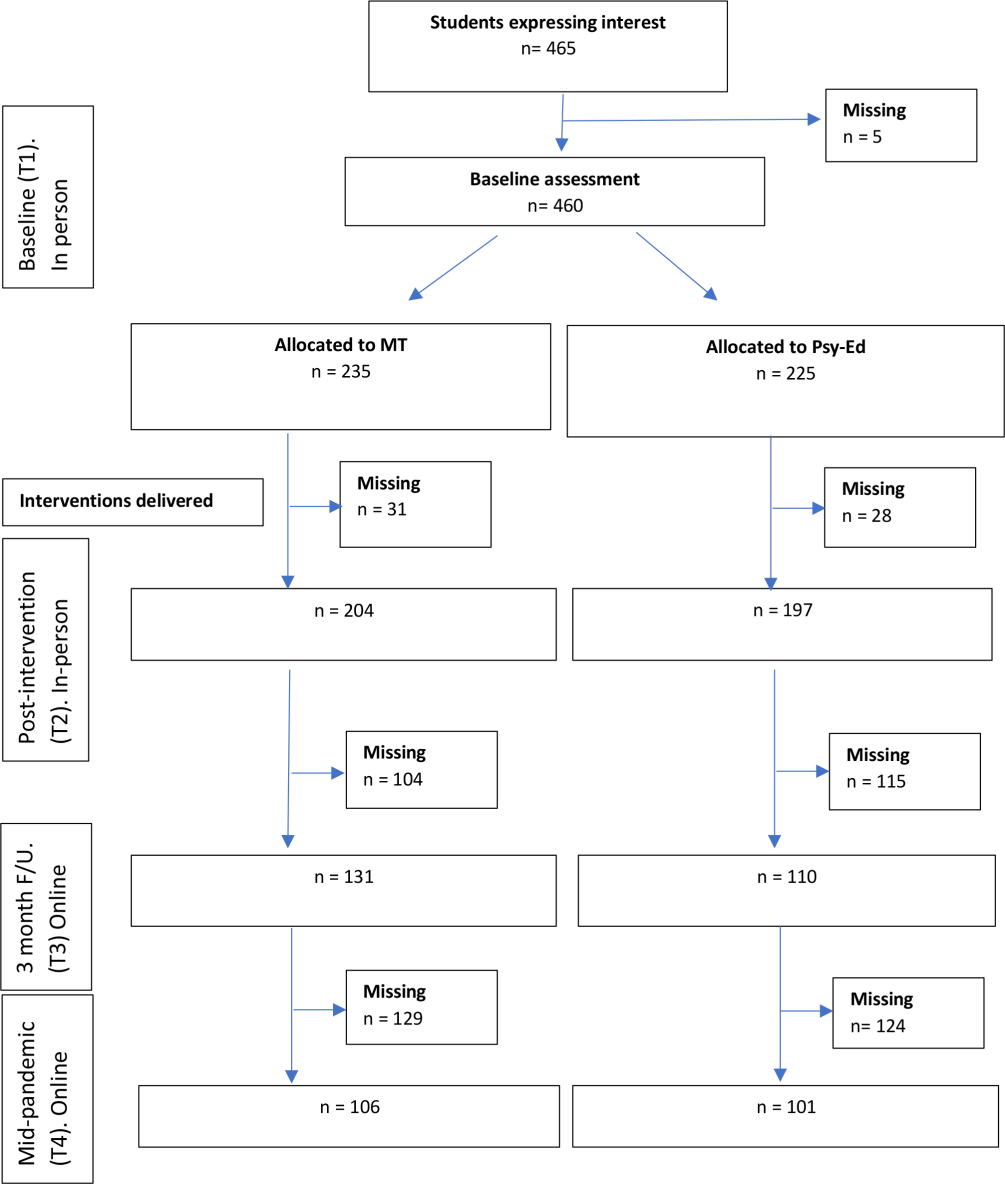
CONSORT flow chart for trial. MT, mindfulness training.

Participant flow is described in the Consolidated Standards of Reporting Trials (CONSORT) diagram (figure 1). Twelve secondary schools from London and Cambridgeshire were recruited. Participants were students aged 11–16 years. All students had the opportunity to take part unless they had a self-reported: (1) Learning difficulty or neurodevelopmental or neurological disorder; (2) Mood disorder currently in episode. Eligible students were asked to return a signed parental consent form, and places were offered on a first-come-first-served basis until our target sample size was achieved. Assent was obtained from all participants. As this was a mechanisms trial, we sought to maximise the probability that students received a complete dose of the interventions. Participants were therefore paid up to £100 for their contribution, based on session attendance and homework completion, to maximise engagement. See online supplemental A for the payment breakdown, and additional information on school recruitment, and the data collection procedure.

10.1136/ebmental-2022-300460.supp1 Supplementary data

### Randomisation and allocation concealment

After consent and assent forms were received, participants within a school were randomly assigned in a 1:1 ratio to MT or Psy-Ed following the Baseline (T1) assessment. Randomisation, with no stratification, was conducted by a statistician (PW), independent of the research team, in blocks of 10 participants with randomisation within each block applied using a computerised random number generator. Due to the nature of MT and Psy-Ed, participants and teachers were not blind to treatment allocation. Researchers collecting data at the face-to-face testing sessions were blind to intervention allocation.

### Interventions

MT and Psy-Ed are described fully in online supplemental B, including adjustments to each intervention to compile 8-week programmes, and information about the training of intervention teachers. Information on assessment of teacher fidelity and student adherence is presented in online supplemental C.

#### Mindfulness training

The MT programme aimed to teach mindfulness skills^1^ that support young people’s resilience, using a combination of class discussion and mindfulness practice. MT used here was an adapted version of .b (dot-be), a 10-week mindfulness course for 11–18-year-old participants, developed by the Mindfulness in Schools Project.^14^ We selected .b due to the encouraging data on its efficacy on measures of mental ill health and well-being.^15^ The adjusted .b curriculum comprised eight 45 min, weekly MT sessions delivered face-to-face in small groups (10–13 participants), at the students’ school. Participants are asked to complete homework between sessions.

#### Psy-Ed curriculum

Psy-Ed was an adapted version of Student Success Skills^9^ an 8-week course designed to teach cognitive, social and self-management skills. Session length, format, delivery and homework requirements were comparable to MT.

### Outcome measures

All measures are described in detail in online supplemental D. The timetable for their administration is presented in online supplemental table S1.

#### Affective executive control

To evaluate the hypothesised mechanism of affective executive control we administered three computer-administered experimental tasks and three questionnaires at baseline (T1) and postintervention (T2).

#### Affective working memory capacity

This complex span task measures the influence of affective contexts on working memory capacity.^7^ Participants are required to remember lists of neutral words, while simultaneously counting how many distracting shapes appear briefly on the screen. Task valence is manipulated via a background image that is either emotionally neutral or emotionally negative in content. Affective executive control is quantified as the proportion of words correctly recalled in the neutral condition minus the proportion correctly recalled in the negative condition.

#### Affective Stroop

This task measures the inhibitory component of affective executive control.^16^ Participants are presented with series of faces with either happy, neutral or sad expressions. A sad or happy word is superimposed over each face such that the resulting face-word pairs can be either congruent (matched in valence; eg, sad facial expression with a sad word), neutral (where the facial expression is pixelated out) or incongruent. Participants are required to categorise the words as ‘happy’ or ‘sad’ as quickly and accurately as possible, while trying to ignore the facial expression. Affective executive control is quantified as the reaction time (RT) to respond in the incongruent condition minus the RT for the congruent condition.

#### Affective sustained attention to response task (aSART)

This measures sustained attention in the face of affective distraction. Participants are presented with a stream of 540 single digits, one at a time. They are required to press a button as fast as possible for every digit unless it is a ‘3’ (the target) when they must withhold the button press.^17^ The digits are presented against a soundtrack that is either emotionally neutral or emotionally negative. The two indices of affective executive control are the number of commission errors (incorrectly pressing the button when presented with the target) and the RT variance for correct responses, with performance in the neutral condition subtracted from performance in the negative condition.

#### Questionnaires

Participants also completed: the Behaviour Rating Inventory of Executive Function (BRIEF^18^) as a self-report measure of executive control; the Child-Adolescent Mindfulness Measure^19^ to assess mindfulness skills; and the Difficulties in Emotion Regulation Scale (DERS^20^) to assess emotion regulation problems.

#### Mental health outcomes

We used three primary mental health outcomes in line with the MYRIAD pragmatic trial:^21^ symptoms of depression (Center for Epidemiological Studies Depression Scale^22^); social, emotional and behavioural functioning (Strengths and Difficulties Questionnaire Total score, SDQ-Total^23^); and well-being (Warwick-Edinburgh Mental Well-being Scale^24^).

We included a range of secondary mental health outcomes: the subscales of the SDQ; anxiety (total score and subscales from the Revised Child Anxiety and Depression Scale^25^) and rumination (Ruminative Response Scale^26^).

### Statistical analysis

The target sample size of 450 students (225 students per trial arm) for the mechanisms RCT was calculated to detect a difference of 0.3 SD units (effect size) on any given affective control outcome measure, with 80% power, at an uncorrected 5% (two-sided) level of significance, assuming 20% attrition at postintervention (T2).

Participant characteristics were summarised using means and SDs for continuous variables and numbers and percentages for categorical variables. Outcomes were compared between trial arms using the intention-to-treat principle. If participants missed a subset of >20% of items for a given measure the outcome was counted as missing. Missing outcome data (assumed to be missing at random) were subject to multiple imputation using the ‘pan’ package in R^27^ (online supplemental E). Sensitivity analyses using complete case data were also undertaken for each outcome. Outcomes were compared between trial arms using mixed-effects (‘multilevel’) linear regression models, specifying a random intercept for school, adjusting for student gender and baseline (T1) score on the outcome.

We also explored the moderating effects of age and gender on the relationship between intervention received and our primary mechanisms outcomes at postintervention. Outcome analyses used the ‘mi’ suite of commands in Stata V.16.1.

Planned analyses of the putative mediating effect of affective executive control on the relationship between intervention received and mental health outcomes were dependent on robust effects of MT versus Psy-Ed on affective executive control.^10^ For the mediation analysis plan, see online supplemental F.

## Results

Table 1 shows descriptive statistics for the MT and Psy-Ed groups at baseline (T1) on the primary affective executive control and mental health outcomes. For secondary mental health outcomes and additional affective executive control metrics at baseline, see online supplemental table S2 and S3, respectively.

**Table 1:** Baseline (T1) characteristics by trial arm status

	MT N=235		Psy-Ed N=225	
	N	%/mean (SD)	N	%/mean (SD)
Location				
London	168	71.5%	160	70.8%
Cambridge	67	28.5%	65	28.8%
Female	155	66.0%	151	67.1%
Age	235	13.8 (1.3)	225	13.9 (1.4)
IQ	221	110.6 (19.0)	218	109.5 (16.0)
CES-D	232	16.3 (9.9)	221	16.6 (10.3)
SDQ-Total	219	12.4 (5.7)	208	13.1 (5.6)
WEMWBS	232	47.1 (10.1)	222	47.2 (9.3)
CAMM-Total	233	25.2 (8.0)	221	24.1 (7.9)
BRIEF-Total	212	133.3 (24.6)	194	136.4 (24.4)
DERS-Total	224	85.4 (23.2)	217	88.3 (26.1)
aSART-C	193	0.5 (3.7)	191	0.2 (4.0)
aSART-RTV	193	0.01 (0.1)	192	0.01 (0.1)
aSTROOP	203	63.4 (93.2)	195	61.8 (102.4)
aWMC	219	0.02 (0.2)	205	0.03 (0.2)

aSART-C was computed as the number of errors in the negative condition minus the number in the neutral condition. aSART-C was reliably different to zero (directional 1-sample t-test) at baseline, *t*=1.79, *p*=.039. aSART-RTV was computed as RTV in negative condition minus the RTV in the neutral condition. aSART-RTV was reliably different to zero at baseline, *t*=2.89, *p*=.004. aStroop was computed as mean reaction time in the incongruent trials minus mean reaction time in the congruent trials. aStroop was reliably different to zero at baseline, *t*=12.78, *p*<.0001. aWMC was computed as the proportion of correctly remembered words in the neutral condition minus the proportion remembered in the negative condition. aWMC was reliably different to zero at baseline, *t*=3.06, *p*=.002.

aSART-C, Affective Sustained Attention to Response Task Commission error; aSART-RTV, Affective Sustained Attention to Response Task Reaction Time Variance; aStroop, Affective Stroop performance; aWMC, Affective Working Memory Capacity; BRIEF-Total, Behaviour Rating Inventory of Executive Function Version 2 Global Composite Score; CAMM-Total, Child and Adolescent Mindfulness Measure Total Score; CES-D, Center for Epidemiological Studies Depression Scale; DERS-Total, Difficulties with Emotion Regulation Scale Total Score; IQ, score on the Cattell Culture Fair Test^29^; MT, Mindfulness Training; Psy-Ed, psychoeducation training; SDQ-Total, Strengths and Difficulties Questionnaire Total Score; WEMWBS, Warwick-Edinburgh Mental Well-Being Scale.

At baseline, the three experimental tasks produced reliable decrements in performance in affective conditions relative to neutral conditions testifying to their validity as indices of affective executive control (table 1). There were also the expected relationships between underlying (non-affective) task performance (indexed by performance in the neutral task conditions only) and age and IQ, with performance being better with older age and higher IQ (online supplemental table S4). Finally, scores on several affective executive control measures were lower at baseline in individuals with higher levels of mental health difficulties. These relationships were stronger and more consistent for the self-report measures than for the tasks (online supplemental table S4).

Of students recruited to the trial, 87.2% (86.8% in MT; 87.5% in Psy-Ed) were retained at the primary postintervention end point (T2) for affective executive control outcomes. Students retained at T2 were younger, and less likely to be female or from a London school, relative to those lost to follow-up (online supplemental table S5). Of the recruited students, 45% (45.1% in MT; 44.8% in Psy-Ed) were retained at the mid-pandemic assessment (T4), the primary end point for the mid-pandemic mental health outcomes. Given that this T4 assessment was an unplanned trial extension, these lower retention rates are perhaps unsurprising. Students retained at T4 had a lower proportion from London schools relative to those lost to follow-up (online supplemental table S6).

### Intervention dose, quality and fidelity

The two interventions were delivered as planned. Students received an average of 6.4 (SD=2.5) sessions of MT and 6.4 (SD=2.3) of Psy-Ed, out of a possible 8. Homework was scored as completed if students self-reported watching the homework video and carrying out the related exercises for that week, and also reported some daily practice of exercises or techniques from the previous lesson. Students reported completing an average of 5.1 (SD=2.3) weeks of homework assignments for MT and 4.5 (SD=2.3) for Psy-Ed, out of a possible 7. Remunerating students for homework completion appeared to enhance engagement when compared with our larger trial where homework was not remunerated and where students reported practising on average much less.^21^ On average, teachers were rated as delivering the intervention with high fidelity (MT=95.4% curriculum adherence; Psy-Ed=94.5%).

### Trial outcomes

#### Affective executive control outcomes

The primary affective executive control outcomes at postintervention (T2) are reported in table 2. There was no evidence of a difference between arms on any outcome. The conclusions of the sensitivity analyses using complete cases were robust to those of these primary analyses. There was no evidence of any moderating effects of age and gender on the relationship between intervention received and these affective control outcomes at postintervention (online supplemental table S7). For descriptive data and outcomes for the remaining experimental task metrics and the BRIEF and DERS subscales at T2, see online supplemental table S8. There was no evidence of a difference between arms on any of these additional variables. Because there was no support for an effect of intervention arm on any affective control outcomes, follow-up mediation analyses that presuppose such effects were not indicated,^10^ despite some evidence that changes in affective executive control (on the DERS, the BRIEF and the affective sustained attention to response task) related to changes in mental health across the intervention period (online supplemental table S9).

**Table 2:** Primary affective executive control outcomes at postintervention (T2)

	MT		Psy-Ed		Unadjusted (I–C)	Adjusted (I-C)		
	N	Mean (SD)	N	Mean (SD)	Mean diff.	Mean diff.	95% CI	P value
CAMM-Total	204	24.8 (7.8)	195	24.2 (8.2)	−0.08	−0.1	−1.7 to 1.4	0.86
BRIEF-Total	187	133.3 (25.4)	175	136.9 (26.8)	−4.4	−2.5	−6.9 to 1.9	0.27
DERS-Total	200	85.8 (23.4)	194	87.1 (25.8)	0.8	1.0	−3.8 to 5.7	0.69
aSART-C	168	−0.1 (4.0)	157	0.2 (4.1)	−0.3	−0.3	−1.4 to 0.7	0.52
aSART-RTV	168	−0.005 (0.1)	158	−0.007 (0.1)	−0.004	−0.01	−0.1 to 0.1	0.90
aSTROOP	172	38.1 (72.0)	162	54.3 (111.5)	−18.9	−20.2	−41.7 to 1.4	0.07
aWMC	148	−0.02 (0.1)	156	−0.02 (0.1)	0.02	0.02	−0.1 to 0.1	0.61

Data presented are complete cases. Inferential statistics are on the full imputed data set (n=460). aSART-C was computed as the number of errors in the negative condition minus the number in the neutral condition. aSART-RTV was computed as RTV in negative condition minus the RTV in the neutral condition. aStroop was computed as mean reaction time in the incongruent trials minus mean reaction time in the congruent trials. aWMC was computed as the proportion of correctly remembered words in the neutral condition minus the proportion remembered in the negative condition.

aSART-C, Affective Sustained Attention to Response Task Commission; aSART-RTV, Affective Sustained Attention to Response Task Reaction Time Variance; aStroop, Affective Stroop Performance; aWMC, Affective Working Memory Capacity; BRIEF-Total, Behaviour Rating Inventory of Executive Function version 2 Global Composite Score; C, Control (Psy-Ed); CAMM-Total, Child and Adolescent Mindfulness Measure Total Score; DERS-Total, Difficulties with Emotion Regulation Scale Total Score; I, Intervention (MT); MT, mindfulness training; Psy-Ed, psychoeducation training.

#### Mental health outcomes

The primary mental health outcomes are reported at the relevant end point (T4) to assess mid-pandemic mental health in table 3, along with outcomes at postintervention (T2). Secondary mental health outcomes are in online supplemental table S10. For completeness, the additional 3-month assessment (T3) mental health outcomes are in online supplemental table S11.

**Table 3:** Primary mental health outcomes at the mid-pandemic primary end point (T4) and at postintervention (T2)

	Time point	MT		Psy-Ed		Unadjusted (I–C)	Adjusted (I-C)		
		N	Mean (SD)	N	Mean (SD)	Mean diff.	Mean diff.	95% CI	P value
CES-D	Postintervention	204	15.9 (9.8)	196	16.9 (10.9)	−1.2	−1.1	−3.1 to 0.9	0.27
	Mid-pandemic	106	20.8 (11.4)	101	21.2 (12.7)	−0.1	−0.09	−2.9 to 2.8	0.95
SDQ-Total	Postintervention	203	11.8 (5.7)	196	13.0 (5.9)	−0.8	−0.8	−1.9 to 0.2	0.13
	Mid-pandemic	104	13.3 (4.9)	101	13.3 (6.0)	0.8	0.8	−0.7 to 2.4	0.28
WEMWBS	Postintervention	203	48.0 (9.8)	196	47.7 (10.1)	1.0	0.9	−0.9 to 2.7	0.34
	Mid-pandemic	106	44.6 (8.9)	101	45.2 (11.2)	−0.002	−0.08	−2.6 to 2.4	0.95

Data presented are complete cases. Inferential statistics are on the full imputed data set (n=460).

C, Control (Psy-Ed); CES-D, Center for Epidemiological Studies Depression Scale; I, Intervention (MT); MT, mindfulness training; Psy-Ed, psychoeducation training; SDQ-Total, Strengths and Difficulties Questionnaire Total Score; WEMWBS, Warwick-Edinburgh Mental Well-Being Scale.

Student mental health across all outcomes was worse at mid-pandemic (T4), relative to the prepandemic, postintervention (T2) assessment (table 3; online supplemental table S10). However, there was no evidence of a difference between arms at mid-pandemic (T4) for any primary or secondary mental health outcome, with the exception of the secondary outcome of the Conduct Disorder subscale of the SDQ for which scores were lower (indicative of better mental health) in the Psy-Ed arm. The conclusions of the sensitivity analyses of complete cases were robust to those of the primary analyses. The pattern of findings was similar at postintervention (T2) with no significant differences between arms on any mental health outcome (table 3; online supplemental table S10).

### Adverse events

For the duration of the interventions, students were advised that they could report any problems or unwanted effects in relation to either intervention to their external intervention teacher or to their internal classroom teacher. We also asked students at the end of the intervention about their experiences, including any unwanted effects. No adverse events were reported.

## Discussion

This randomised controlled mechanisms trial evaluated the impact of a widely used MT programme, relative to a Psy-Ed control intervention, in early adolescence on experimental-tasks and self-report measures of the key proposed mechanism-of-action of MT—affective executive control. There was no evidence for a superior impact of MT, relative to Psy-Ed, on any measure of affective executive control. The mechanisms trial was completed just prior to the onset of the worldwide COVID-19 pandemic. We therefore took the opportunity to extend the trial to a mid-pandemic assessment following the first UK national lockdown in summer 2020 to evaluate the hypothesis that MT, relative to Psy-Ed, would mitigate against detrimental effects of the pandemic on mental health. We found no evidence to support this hypothesis.

It is always difficult to interpret null findings and conclusions must therefore be tentative. There are a number of possible explanations for the null findings with respect to affective executive control. In the absence of a no intervention or passive control condition it is not possible to draw definitive conclusions on which of these explanations is most valid. First, it may be that either MT does not have a differential effect on this proposed mechanism of action, or that both MT and Psy-Ed affect this mechanism, or that the effect is delayed beyond the postintervention time point, or that it affects subgroups differently.^28^ Second, it may be that the version of MT used here—.b—is actually benign and does not deliver sufficient training in key mindfulness skills to impact our outcomes. We selected .b due to its encouraging results in our pilot trial.^15^ The intervention was also delivered with high fidelity by experienced external teachers, and we ensured good engagement from participants through financial compensation. However, in our subsequent large-scale pragmatic trial^21^ we found no support for .b on any mental health outcome measure. Finally, it may be that our affective control outcomes were insensitive measures of control. This seems unlikely to be uniformly true as they comprised a broad set of widely used measures, both experimental and self-report, including a measure of mindfulness skills overlapping closely in content with the content of the .b curriculum.

In terms of the mid-pandemic mental health outcomes, it may be that both MT and Psy-Ed are efficacious or that neither is efficacious. Alternatively, it may be that MT offers protection for different outcomes than those used here, or only when the intervention is delivered closer in time to the stressor.

The study has a number of noteworthy strengths. The trial was adequately powered to address its primary mechanisms question. MT was delivered with good fidelity by experienced teachers. There was good engagement from students and we had good retention at the primary mechanisms end point (87%). We used a mix and range of self-report and experimental measures of affective executive control and a comprehensive set of mental health outcomes.

The study also had a number of limitations. Students and teachers were necessarily not blind to treatment allocation. We did not include a no intervention or passive control condition limiting the conclusions that can be drawn from the present pattern of null findings. We did not include a follow-up assessment for the affective executive control measures. Finally, for the unscheduled COVID-related mental health trial extension, retention rates were perhaps unsurprisingly low, and there was a wide range of follow-up duration from the trial baseline.

## Conclusions

In a rigorous, randomised controlled mechanisms trial we found no support for our hypothesis that MT would have a greater postintervention impact on affective executive control relative to Psy-Ed. In a trial extension, we found no support for MT conferring protection against deterioration in mental health following the onset of the COVID-19 pandemic relative to Psy-Ed. It may be that MT has no impact on affective executive control or stressor-related mental health, that both MT and Psy-Ed have a comparable impact, or that the version of MT deployed here—.b—was benign. In the absence of additional control conditions definitive conclusions are not possible.

## Data Availability

Data are available upon reasonable request. The data and codebook for the MYRIAD Trial are available from the corresponding author upon request (release of data is subject to an approved proposal and a signed data access agreement).
