# Supplementary material for: The impact of mindfulness training in early adolescence on affective executive control, and on later mental health during the COVID-19 pandemic: a randomised controlled trial
Source: Evid Based Ment Health. 2022 Jul 7;25(3):110–6. doi: 10.1136/ebmental-2022-300460 (PMC9340025; doi:10.1136/ebmental-2022-300460)
Supplement: Supplementary data [file ebmental-2022-300460supp001.pdf]

The impact of mindfulness training in early adolescence on affective executive control, and on later mental health during-COVID-19: A randomised controlled trial

Supplements and supplementary tables

Supplement A: School, teacher and student recruitment details and data collection procedure

Supplement B: Descriptions of the interventions and training of the intervention teachers

Supplement C: Assessment of adherence and fidelity

Supplement D: Detailed description of study measures

Figure S1: A sample 4-trial block for the Affective Picture Span Paradigm (APSP) for the Negative condition, adapted from [1].

Figure S2: Example of an aSART trial sequence

Figure S3: Examples of Congruent, Neutral and Incongruent Stimuli from the Affective Stroop Task

Supplement E. Multiple imputation model.

Supplement F: Mediation analysis plan

Table S1: Primary and secondary mental health and affective cognitive control outcomes across the four trial time points.

Table S2: Secondary mental health outcomes at baseline

Table S3: Additional cognitive task variables at baseline

Table S4: Zero-order Pearson correlations between key affective control variables and the primary mental health outcomes at trial Baseline (T1)

Table S5: Differences between complete cases and whole sample at post-intervention (T2)

Table S6: Differences between complete cases and whole sample at mid-pandemic (T4)

Table S7: Moderating effects of age and gender on the relationship between intervention received and the primary mechanisms outcomes at post-intervention (T2)

Table S8: Additional affective executive control variables at post-intervention (T2)

Table S9: Zero-order Pearson correlations between pre- to post-intervention change (Δ)in primary affective control variables and in mental health outcomes

Table S10: Secondary mental health outcomes at the mid-pandemic primary endpoint (T4) and at post-intervention (T2)

Table S11: Primary and secondary mental health outcomes at the additional 3-month follow up assessment (T3)

Supplement A: School, teacher and student recruitment details and data collection procedure

Schools. Twelve secondary schools were recruited in London and Cambridge. Schools agreed to randomisation, to external teachers delivering the MT and Psy-Ed curricula on-site after school hours, and to providing space for the pre- and post-intervention data collection sessions. Schools were recruited via several routes. Firstly, schools that had taken part in previous research by the authors. Secondly, other large schools in London and Cambridge were contacted. We did not include special schools, alternative provision settings, or schools that currently teach mindfulness to all students. Schools that teach some mindfulness were still eligible, provided that mindfulness had never been taught to the students who will take part in the current study.

Students. Within schools, students aged 11-16 were eligible to take part. For each school, two year groups were selected based on a discussion between the research team and the school. Specifically, the research team requested specific year groups in order to cover that age range (for example, the research team may have requested a group of Year 9 students in order to ensure a sufficient number of 13 and 14 year olds), and the school would advise on whether their students of that age are available to take part in the proposed term. The school may restrict access to students depending on other commitments: for example, they may request that Year 7 students do not take part in the autumn term as they have just started a new school, or that Year 11 students during the summer term due to GCSE examinations. Up to two consecutive year groups (e.g., Years 8 and 9) would take part together if this is appropriate for the research team and convenient for the school.

To mitigate risk of selection bias, all students in selected year group/s were offered the opportunity to take part, with two exceptions. Firstly, students with a diagnosis of a neurodevelopmental or neurological disorder or learning difficulty were not be eligible to take part, due to the possibility that their diagnosis will affect the way in which they complete the battery of measures. Secondly, students with a recent self-disclosed trauma were not be eligible to take part, due to the potential risk of harm of taking part in the reflective process of mindfulness training. Students with psychiatric diagnoses were eligible to take part, provided the above two criteria were met.

Standard emails, written by the research team, were then sent to parents/caregivers of all students in year groups selected as eligible by the school and research team. These included an information sheet with details of the study, and a consent form, giving them the opportunity to opt in to the research project for their child. Students with returned consent forms were selected to participate in the study on a first-come first-served basis.

For students selected to participate in the study, emails were sent to parents/caregivers from the school reminding them of the next step for their child. The same letter/email was sent to all parents/caregivers irrespective of the intervention to which the child was to be randomised. Students aged 16 and over also provided their own consent. All students also provided assent.

For students not selected (i.e. those whose consent forms were not returned early enough, and who therefore were not selected to take part), letters were sent to parents/caregivers from the school informing them that their child's involvement in the study has ended.

Data collection procedure. Following informed consent, participants within each school were randomly assigned to MT or Psy-Ed. This randomisation was conducted by a statistician (PW) independent of the research team.

Dunning D, *et al. Evid Based Ment Health* 2022; 25:110–116. doi: 10.1136/ebmental-2022-300460

Before (T1) and after (T2) the interventions, all participants attended two data collection sessions in which they completed a battery of questionnaires and experimental tasks. Participants also completed online follow-up questionnaires at three months follow-up (T3) and a final series of online questionnaire at between 20 and 44 months post-intervention after the first UK lockdown (June-July 2020) as a result of the COVID-19 pandemic (T4). The T1 and T2 data collection sections were each divided into two after-school sessions, each lasting 1.5 hours.

Participants were compensated £15 for each of the two group data collection sessions; £5 per classroom session attended, contingent upon completion of the home practice sheet for that week; a £10 bonus for good attendance of the training sessions and completion of both group data collection sessions; a £10 voucher upon completion of the additional 3-month online data collection session; and a £10 voucher for completion of the mid-pandemic T3 online assessment, amounting to a possible £100 in total.

Intervention teachers. Intervention teachers were existing MT teachers with experience of teaching at least one .b course, alongside additional experience teaching at least one other social and emotional learning (SEL) curriculum. Intervention teachers were required to teach both the MT and the Psy-Ed curricula.

Supplement B: Descriptions of the interventions and training of the intervention teachers

MT curriculum. The MT curriculum is a slightly adapted version of .b (dot-be), a 10-week mindfulness course for 11-18 year olds, developed by the Mindfulness in Schools Project (MISP) in the UK. The goal of the MT curriculum is to enable adolescents to learn mindfulness skills, which involves training one's attention to focus on the present moment, with an attitude of non-judgemental acceptance. The MT curriculum was drawn primarily from mindfulness-based stress reduction and mindfulness-based cognitive therapy.

There are ten lessons in the original version of .b: Introduction; Paying Attention; Taming the Animal Mind; Moving Mindfully; Stepping Back; Befriending the Difficult; Taking in the Good; and Pulling it All Together. Each lesson is forty-five minutes long and is taught with an accompanying slideshow presentation. Each slideshow presentation is animated and includes short video clips. Participants are also given a homework sheet each week, tailored to the content of that week's lesson. As part of the homework, participants are requested to practice mindfulness using animated videos online. We asked participants questions on the video content to ensure compliance. Students were also asked to complete daily practice of one of the components of the previous lesson and record their practice on a homework sheet.

In the current study, the ten lessons were adapted to form an eight-week course. This was to allow the intervention and the pre- and post- intervention (Time 1 and Time 2) data collection sessions to be completed within a single academic school term within the UK. To achieve this, two changes were made to the original curriculum. Firstly, the Introductory and second (Lesson One – Paying Attention) lessons were combined. Secondly, Lesson Five (Mindful Movement) was removed. These adaptations were made on the recommendation of existing curriculum teachers and the curriculum developers as they were deemed to be those least likely to impact on the key principles of the programme.

Psy-Ed curriculum. The Psy-Ed curriculum was an adapted version of Student Success Skills (SSS), an eight-week course developed in the USA. The programme was designed to help students develop key cognitive, social and self-management skills.

Each of the eight lessons was taught with a PowerPoint presentation. Five adaptations were made to the original material for inclusion in this study. Firstly, the original eight-lesson course was made up of a six-lesson course delivered over six consecutive weeks, with two 'booster' lessons that were given after one month and two months respectively. In the current study, the same content was delivered over eight consecutive weeks. Secondly, in the original intervention, the two booster lessons (Lessons 7 and 8) were identical to Lesson 6. In the current study, because Lessons 6, 7 and 8 were being taught in three consecutive weeks, the content of Lessons 7 and 8 were adapted in order to provide variation. The new material added to these two sessions was in keeping with the original themes and messages of the intervention. Thirdly, the slides were modernised and adapted for a UK student audience. Specifically, images and fonts were updated and videos and animations were added, in order to match visual appeal across the two interventions. Adaptations were assessed with a focus group. The fourth adaptation was that some content was removed from the intervention because it was deemed to be too similar to mindfulness, as agreed by researchers with expertise in mindfulness. Finally, participants were also given a homework sheet each week, tailored to the content of that week's lesson. The original programme included tasks for students to complete in their own time throughout the week, but we designed homework sheets to accompany these tasks so that we could have a record of the homework done, and also to match the homework content of the MT curriculum.

Intervention training for teachers. All teachers attended a two-day workshop prior to taking part in the study. This workshop covered three aspects of training: an introduction to the study; the adaptations to the MT intervention used in the current study, and the Psy-Ed intervention. In the introduction to the study section, it was emphasised that no aspect of mindfulness should be taught in the Psy-Ed curriculum, and also the importance of teaching both interventions with equal enthusiasm and engagement was stressed. Teachers were paid £250 for their attendance at the training and £1000 for running each 8-session interventions.

Supplement C: Assessment of adherence and fidelity

Student adherence was assessed in two ways. First, attendance at sessions was recorded. Secondly, students were required to record their home practice on a series of homework sheets.

The fidelity of the intervention's teaching was assessed via video recording. Each group had one lesson recorded at random. For ethical reasons, only the teacher (and not the students) was visible in the recording. An independent assessor who was a qualified classroom teacher and who had several years of experience in in delivering both MT and Psy-Ed courses, rated videotapes of MT and Psy-Ed classes for fidelity

A single session from each course was randomly selected using a random number generator for filming. Teachers did not know in advance that which lesson would be chosen but were provided with several days' notice that the filming would take place. For each lesson observed, independent evaluators indicated whether key curriculum elements (essential and non-essential, as they are defined by the MT and SST teaching materials), were delivered or not. These ratings were summarised as the percentage of curriculum elements covered per each lesson.

Dunning D*, et al. Evid Based Ment Health* 2022; 25:110–116. doi: 10.1136/ebmental-2022-300460

Supplement D: Detailed description of study measures

Affective Working Memory Capacity (aWMC). AWMC is a measure of the influence of an affective context on (short term) working memory in the presence of distractors. AWMC was assessed here using an Affective Picture Span Paradigm (APSP) adapted from a version used in a previous study in adults [1]. The task comprised two cognitively engaging components. The first is a target (storage) task, where participants are told to learn and retain a set of words, presented one word per trial in blocks of 2-5 trials and superimposed upon a background image. All images were taken from web depositories including Freerange, Pixbay, Unsplash, Pexels, StockSnap, that are copyright and royalty free. The second component is the operation (distractor) task, which was interpolated with the target task, and involved a varying number of shapes (pink squares) appearing over the same background image on each trial, before and after the target word is presented, with the number of shapes varying in the range 4 to 6 across trials. Participants were instructed to count and report the number of shapes within a given trial at the same time as memorising the word on that trial.

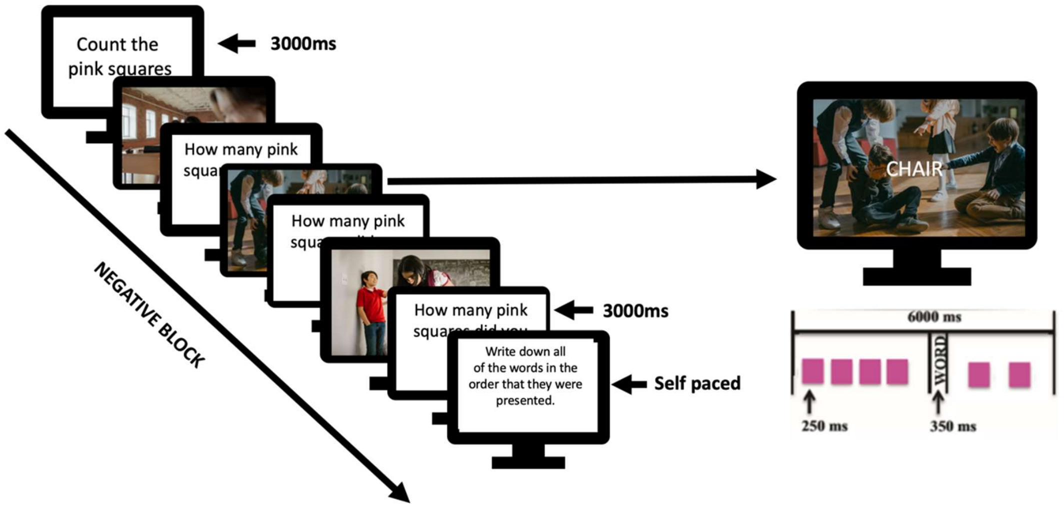

Figure S1. A sample 3-trial block for the Affective Picture Span Paradigm (APSP) for the Negative condition, adapted from [1].
All images are copyright and royalty free and taken from https://www.pexels.com/. The license information for these images is here: https://www.pexels.com/license/
The individual image addresses (from top left in the figure) are:

https://images.pexels.com/photos/7396509/pexels-photo-7396509.jpeg?auto=compress&cs=tinysrgb&w=1260&h=750&dpr=2

https://images.pexels.com/photos/7929446/pexels-photo-7929446.jpeg?auto=compress&cs=tinysrgb&w=1260&h=750&dpr=2

https://images.pexels.com/photos/6936338/pexels-photo-6936338.jpeg?auto=compress&cs=tinysrgb&w=1260&h=750&dpr=2

At the start of each trial, 2-3 shapes appeared one-at-a-time and sporadically over the background image (see Figure S1). Next, the to-be-remembered word for that trial appeared over the image. Finally, the second set of 2-3 shapes appeared. The numbers of shapes in a given trial (maximum of 6) was randomly set before and after the presentation of the target word. The screen, including the image, then cleared, and participants were prompted to input the number of target shapes they had seen using the keyboard.

Trial blocks comprised between two and five trials. At the end of a trial block, participants were asked to type as many of the presented words as they could remember in the temporal (trial) order in which they had been presented. There was no time restriction on recall. The recall screen presented one blank box per word to cue participants on how many words had been presented.

Task valence was manipulated by presenting background socially-relevant images that were either emotionally Neutral or emotionally Negative in content. Images depicting negative social-contexts (such as bullying or social exclusion) were rated by an independent group of adolescents for: (i) emotional arousal; and (ii) negative valence, using Self-Assessment Manikins. Significant differences in valence and arousal were found between the emotionally neutral and negative backgrounds.

Each block length (of between 2-5 words) was presented twice in each affective condition (negative, neutral), giving a total of 56 trials over 16 blocks. The neutral blocks and the negative blocks were divided into two sets of eight with the presentation order counterbalanced across participants. After completing the first set of eight blocks, participants had a brief break before they continued with the second set. The whole task took approximately 15 minutes to complete and was programmed using E-prime® 2.0 SP2 and presented on 13-inch laptops. Before the task, participants read the instructions on the screen and completed two practice trials. Participants had the opportunity to ask any questions before the task commenced.

To reduce floor effects in this younger population, adjustments were made to the adult version of the APSP, these were: (i) Reducing the maximum block size from 7 to 5 trials, (ii) using only one type of target shape at a time for the operation component, (iii) adding slots to the recall screen matching the number of words presented, (iv) reducing the proportion of operation task trials that needed to be correct for the participant's data to be retained (the Attention Target Detection Rate; ATDR) to 42.75%. The task was also modified to encourage group-based data collection through the use of written recall (rather than verbal). This minimised peer distraction and encouraged individuals to complete the task in exam-style conditions.

To compute separate WMC scores for the Negative and Neutral conditions, we calculated the proportion of all words that were recalled in the correct order of presentation (Conway et al. 2005), summed across the 8 blocks in each condition. We then computed an aWMC-index which was computed in percentage terms as the increment in APSP performance for the Neutral condition relative to the Negative condition. Increasingly positive WMC-indices therefore indicate the extent to which participants performed better in the Neutral condition.

Affective Sustained Attention to Response Test (aSART). The aSART measures sustained attention in the face of affective distraction. The aSART was programmed in E-Prime version 2.0. The original version of the SART [2] was designed as a simple, controlled, reliable and

valid measure of lapses in sustained attention. The aSART adapts the original SART through the introduction of different auditory background stimuli – affective versus neutral – to evaluate whether attentional lapses vary as a function of affective context. Apart from the addition of background stimuli, the aSART was identical to the original SART. Both were computer-administered tasks that involved the withholding of key presses to rare (one in nine) targets presented on the screen. Specifically, targets were drawn from the numbers 1-9 and were presented one digit at a time. The participant was simply asked to respond to the appearance of each digit by pressing the space bar ('Go' trials). The exception to this was when the number '3' appeared, to which no response should be made ('No-Go' trials). For the aSART, the response window was 1150 ms (each digit was on screen for 400ms, followed by a mask (a fixation cross) for 750 ms; see Figure S2). Five-hundred and forty trials were presented, 60 of which were 'No-Go' trials, over a period of 12 mins.

Figure S2: Example of an aSART trial sequence

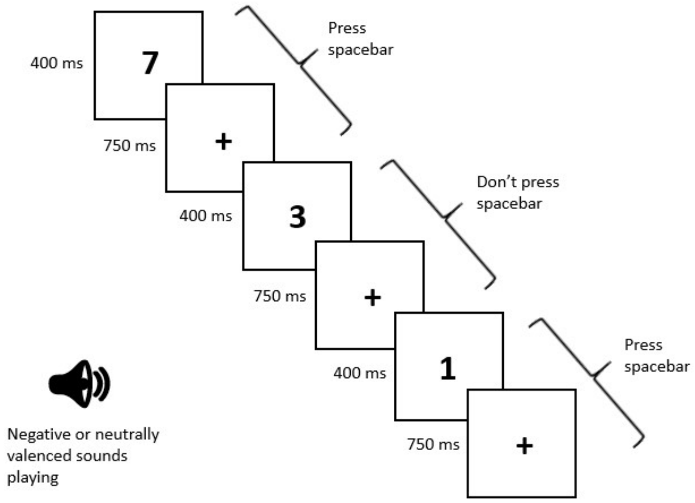

While completing the task, in a within-subjects design, participants listened to a continuous background stream of either neutral- or negative-valence sounds through headphones. The 540 trials were divided into six blocks of 90 trials each. In three of the blocks, participants heard a stream of negative sounds (e.g., an alarm clock going off, a baby crying) and in the other three blocks they heard a stream of affectively neutral sounds (e.g., crowd murmur, farmyard animals). The sounds were from the International Affective Digitized Sounds (IADS) corpus (a library of sounds pre-rated for valence and arousal by college attending adults; [3]). Each sound file lasted approx. 6 seconds. The files were concatenated using a custom script written in MATLAB 2014a. Because the sound files (.wavs) were recorded at various sample rates (8 to 44.1 kHz) they were first resampled to 44.1 kHz in MATLAB to standardise presentation. The respective sets of sounds produced six auditory streams (three negative, three neutral), each lasting approx. 2 mins. Within condition, these were played to participants over headphones in a random order.

The dependent variables for the aSART were as follows: Commission errors were the total number of space bar responses that occurred following the presentation of the no-go digit, 3. Omission errors were the total number of go trials to which a no response was made before the onset of the next trial (1150 ms). Correct reaction time (RT) was the mean interval (ms) between digit onset and response on 'go' trials. RT variance was calculated separately for

each participant by dividing the standard deviation associated with their mean correct RT by their correct RT (standard deviations of reaction times are generally proportionate to the overall magnitude of the mean RT, this 'coefficient of variation' approach effectively removes the influence of overall RT allowing clearer comparison of differences specifically in variability).

The key outcome variables for the present study were commission errors and RT variance. We computed indices to measure the effect of affective context by subtracting scores on these variables in the neutral condition from scores in the negative condition, such that larger scores represented a bigger influence of affective context.

The Affective Stroop Task (AST). The AST measures the inhibitory component of affective executive control. The AST was adapted from a version developed by Preston and Stansfield (2008)[4] and was programmed in E-Prime. The adaptations made for adolescents involved replacing adult faces with adolescent faces, adding a neutral condition, changing the affective words to more age-appropriate alternatives and simplifying the task so that there were only two response options (happy or sad), rather than three (happy, sad or angry).

Stimuli comprised composite pictures of faces with words. We used 12 pictures of faces (three different facial expression from four different adolescent actors) taken from the National Institute of Mental Health Child Emotional Faces Picture Set (NIMH-ChEFS; [5]). Each actor displayed a face with a happy expression, a sad expression, or the face was neutral. For the neutral condition, rather than present a face with a neutral expression, we used a scrambled image of a face as research suggests a neutral expression is perceived as more similar to a sad expression than a happy one (e.g., [6]).

Each face had a different happy (Cheerful, Glad, Jolly, Joyful) or sad (Gloomy, Upset, Miserable, Hopeless) word superimposed semi-transparently over it, centred vertically on the nose. The two word sets were matched in terms of age of acquisition [7]. We created 8 versions of the 12 faces (each face superimposed with each emotional word), giving 96 different face-word composite pictures in total. Each face-word picture was presented twice giving a total of 192 trials; 64 of these were Congruent trials where the word was superimposed over a face with an expression that matched that word in terms of valence, 64 were neutral trials where a word was superimposed over a scrambled face, and 64 were incongruent trials where the word was superimposed over a face with an expression that was the opposite to the valence of the word. Figure S3 shows examples of each condition.

Participants were instructed to respond to the *word* by indicating if it was a happy or sad word and to ignore the facial expression. They were asked to respond "as quickly and as accurately as possible" by pressing a button on the computer keyboard labelled with an 'S' (for sad) or an 'H' (for happy).

A number of measures of performance were derived. Reaction time (RT) is the time taken in milliseconds (ms) for a correct word categorisation response. Accuracy is the percentage of correct responses. Separate measures of RT, and accuracy were calculated for Congruent, Neutral and Incongruent conditions. The key aStroop outcome was calculated by subtracting performance on incongruent trials from performance on congruent trials.

Figure S3: Examples of Congruent, Neutral and Incongruent Stimuli from the Emotional Stroop Task.
Images are taken from the Mental Health Child Emotional Faces Picture Set (NiMH-ChEFS;[5]). As the original article states "The NIMH-ChEFS is freely available to the scientific community for use" **[5; p.146].** ***The whole stimulus set was initially downloaded from: https://devepi.duhs.duke.edu/nimh-chefs-picture-set/The set has moved and is now available at: https://nyulangone-my.sharepoint.com/personal/jason_chavarria_nyulangone_org/_layouts/15/onedrive.aspx?id=%2Fpersonal%2Fjason%5Fchavarria%5Fnyulangone%5Forg%2FDocuments%2FNIMH%2DChEFS%20Picture%20Set&ga=1***

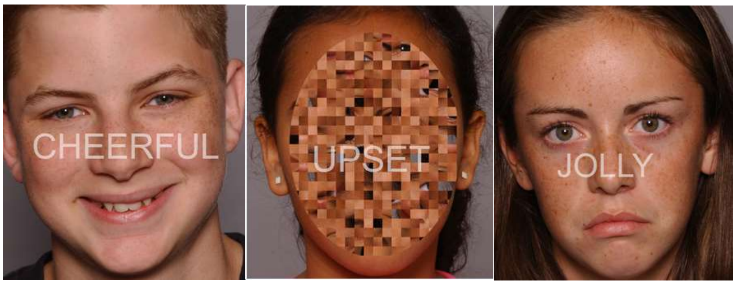

The Cattell Culture Fair Intelligence Test (CCFIT) of verbal IQ. The CCFIT [8] is a measure of non-verbal intelligence that minimises sociocultural and environmental influences. A paper and pencil version of Scale 2 Form A of the test was used at trial Baseline (T1). This comprised four timed subtests consisting of questions involving the relationships between pictures of abstract geometric shapes (e.g., completing a sequence of pictures of shapes or choosing a shape that is different from others). Correct responses were summed, and age-appropriate standard scores were calculated based on a set of existing norms.

Center for Epidemiologic Studies for Depression Scale (CES-D; [9]). The CES-D is a 20-item self-report questionnaire that assesses depressive symptoms in the past week (e.g., "I felt depressed"). It has been validated for use in adolescents [9]. Each item is rated on a rating-scale from 0 ("rarely or none of the time"), to 3 ("most or all of the time"), yielding a total score between 0 and 60, with higher scores indicating greater risk for depression.

Strengths and Difficulties Questionnaire (SDQ, [10]). The SDQ is a 25-item questionnaire that assesses social, emotional, and behavioural strengths and difficulties over the previous 6 months (e.g., "I am constantly fidgeting or squirming"). Each item is rated on a rating-scale from 0 ("not true"), to 2 ("certainly true"). The SDQ's five sub-scales assess emotional symptoms, conduct problems, hyperactivity/inattention, peer problems, and pro-social behaviour. The total difficulties score measures social/emotional/behavioural functioning

Dunning D, *et al. Evid Based Ment Health* 2022; 25:110–116. doi: 10.1136/ebmental-2022-300460

(range 0-40), and is derived by summing the first four subscales, where higher scores indicate greater dysfunctional levels. Student reports of the SDQ were used in the present study.

Warwick-Edinburgh Mental Well-being Scale (WEMWBS; [11]). The WEMWBS is a 14-item measure assessing both feeling and functioning aspects of mental well-being over the last two weeks (e.g., "I've been feeling useful"). Items are scored on a rating-scale from 1, "none of the time" to 5, "all of the time", yielding a total score that ranges between 14 and 70. Items are worded positively and therefore higher scores indicate greater levels of mental well-being.

Behaviour Rating Inventory of Executive Function, Second Edition (BRIEF; [12]). The BRIEF2 is a 55-item self-report measure designed to assess self-perception of everyday behaviours associated with executive function in older children and adolescents (aged 11-18), e.g., "I have trouble sitting still". The BRIEF-2 assesses executive function across the past 6 months and takes into account 7 domains: inhibit; self-monitor; shift; emotional control; task completion; working memory; and plan/organize. Items are rated as follows: 1 = "never", 2 = "sometimes", 3 = "often". Total scores are calculated by summing the sub-scores, with higher scores suggesting higher levels of executive dysfunction. The three items of the infrequency scale ("I forget my name", "I have trouble counting to three", "I cannot find the front door of my home") are only used as indicators of validity and are not included in the calculation of raw scale scores, so that the total score ranges between 52 and 156, and therefore higher scores meaning worse executive functioning. The student-report of the BRIEF was used in the present study.

The Revised Children's Anxiety and Depression Scale (RCADS; [13]). The RCADS is a youth self-report questionnaire that measures separation anxiety disorder (SAD), social phobia (SP), generalized anxiety disorder (GAD), panic disorder (PD), obsessive compulsive disorder (OCD), and major depressive disorder (MDD). For the current project, the questions that make up the depression subscale were removed, to leave a 37-item questionnaire measuring anxiety, asking 'how often' each item happens (e.g., "I worry when I think I have done poorly at something"). Measured using a 4-point Likert-type scale (0 = Never, 1 = Sometimes, 2 = Often, and 3 = Always), the questionnaires subscales are scored by summing the items. A total score can also be calculated by summing the 5 subscales.

The Child and Adolescent Mindfulness Measure (CAMM; [14]). The CAMM; Greco et al., 2010) is a self-report mindfulness skills scale designed specifically for use with children and adolescents. It consists of 10-items, which measure awareness of the present moment as well as non-judgemental and non-avoidant responses to thoughts and feelings (e.g., "I keep myself busy so I don't notice my thoughts or feelings"). Participants are asked how often each sentence is true, and responses are given using a 5-point Likert-type rating-scale ranging from 0, "Never True", to 4, "Always True". Each item is reverse scored and summed, producing a total score of 0-40, with higher scores corresponding to higher levels of mindfulness. The CAMM has been validated for use in non-clinical samples of adolescents, and has adequate psychometric properties [14].

The Difficulties in Emotion Regulation Scale (DERS; [15]). The DERS is a self-assessment scale measuring emotion dysregulation. It includes 36 items scored 1–5 from 1 'almost never' (0–10% of the time), to 5 is 'almost always' (91–100%). The DERS yields a total score as well as six subscales where higher scores indicate more difficulties. The six subscales are *Nonacceptance*, *Goals*, *Impulse*, *Awareness*, *Strategies,* and *Clarity*. The Nonacceptance subscale indexes nonacceptance of emotional responses. Items from the Goals subscale

reflect difficulties engaging in goal directed behaviour. The Impulse subscale measures impulse control difficulties. The Awareness subscale indexes lack of emotional awareness. The Strategies subscale measures limted access to emotion regulation strategies. Finally, the Clarity subscale indexes lack of emotional clarity or a high degree of confusion regarding emotions. The DERS has demonstrated adequate construct and predictive validity as well as good test-retest reliability [15].

The Ruminative Response Scale. The Ruminative Response Scale [16]. The RRS has 22-items that measure how participants typically respond when they "feel down, sad, or depressed" on a scale from 1 (*almost never*) to 4 (*almost always).* It was developed to measure rumination that is related to, but not confounded, by depression. The RRS includes two aspects of rumination, brooding and reflective pondering. A total score can be used, with scores that may range between 22 and 88, with higher scores indicating higher levels of ruminative responses styles. It has been observed that rumination can contribute to more depressive symptomatology. The RRS has demonstrated appropriate factorial structure and psychometric characteristics [17].

Additional tasks. A number of additional bespoke tasks measuring aspects of social functioning were included in the task battery as part of a separate study and are not discussed here. These were as follows:

Social influence task. This task assesses two things. Firstly, how frequently the participant engages in common prosocial (helping) behaviours such as giving up their seat for someone on the bus or antisocial behaviours, such as making fun of a classmate. Secondly, the task assesses the extent to which the participant is influenced by reports of how often other people engage in the same behaviours.

Delay discounting. This task assesses the participant's ability to resist a smaller, immediate reward in favour of a larger reward that requires a wait. The participant is presented with a series of two options, such as receiving £5 today or £10 in two weeks' time, and they must choose which option they would hypothetically prefer.

Sunk cost bias. The sunk cost bias describes the tendency to persist with an investment (financial or other) despite the fact that the costs cannot be recovered and that it is no longer beneficial to continue. This task assesses the participant's ability to resist the sunk cost bias. This task presents the participant with a series of scenarios involving a sunk cost (either a financial cost or one of time/effort) and asks him/her what they would do if faced with that scenario on a 6-point Likert scale (most likely to do X or most likely to do Y, where Y represents resisting the sunk cost bias).

Dictator game. This task assesses the participant's altruistic prosocial behaviour by asking them how they would split a sum of money (£5) between themselves and a charity.

Cyberball. Cyberball is a computer game in which a ball is passed between the participant and two 'online' players (actually programmed by the research team). The task assesses the impact of social exclusion on mood and anxiety. In the game, the participant is sometimes included in the game (the ball is passed to him/her) and sometimes excluded (the ball is primarily passed between the two computer players). Mood and anxiety questionnaires are administered three times: before playing, after being included, and after being excluded.

Dunning D, *et al. Evid Based Ment Health* 2022; 25:110–116. doi: 10.1136/ebmental-2022-300460

Supplement E. Multiple imputation model. Missing outcome data were multiply imputed using the 'pan' package in R.[18] The imputation model included the executive and mental health outcomes, trial arm status, gender, age, location (London/Cambridge), and auxiliary variables of number of intervention sessions attended and amount of homework assignments completed. Fifty imputed datasets were generated, using a multivariate linear mixed effects ("multilevel") model, specifying a random intercept for school. We computed a 'multivariate empty model' where all variables are on the left-hand side of the equation of the imputation model.

Supplement F: Mediation analysis plan. We applied the Kraemer et al. framework for our planned mediation analysis. This required attention to several key aspects of study design [18]. First, MBCT was compared with an intervention that works in terms of its impact on mental health – Psy-Ed – but not through the same proposed mechanism of action, thus allowing a test of effects specific to CT. Second, assessment of change in the hypothesized mediator must occur *during* MT and *before* the assessment of outcome. Finally, those in both intervention arms must receive an adequate dose of the intervention to properly test the hypothesis that MT's impact on the hypothesized mechanisms mediates outcome. We therefore financially compensated participants to maximise adherence.

The Kraemer et al. framework comprises a regression approach in which intervention group (T), the mediator or moderator (M), and the intervention by moderator/mediator interaction term (TxM) are the independent variables. We proposed to examine the mental health outcomes outcome of depressive symptoms using linear regression. Within these regressions, for M to be a mediator of intervention, M must be an event occurring during or after intervention that is significantly altered by intervention and temporally precedes the outcome. **This was in fact the primary trial hypothesis and if this was not supported the mediation analyses would not be possible as the first criterion of an effect of the intervention on the mediator would not have been met.** M must also then show a main and/or interactive effect with intervention on outcome; i.e., the M and/or T x M terms in the regression should be significant. Intervention need not have a significant overall or main effect on outcome [18].

A *main* (but not interactive) effect of mediation is therefore where the intervention significantly changes the mediator but the effect of the mediator on outcome does not significantly differ across intervention types. For example, in the present study if MT differentially improves mindfulness skills or affective executive control and any such improvement translates into better outcome, but the relationship between improvement and outcome does not differ across MT vs. Psy-Ed, this would be a main, but not interactive, effect of mediation.

In contrast, an *interactive* effect of mediation is where the intervention not only significantly changes the mediator but also changes the relationship between the mediator and outcome such that it is significantly different for the alternative interventions. For example, in the present study if intervention significantly affects mindfulness skills or affective executive control, but the relationship between these outcomes and worse mental health is then significantly different in the MT vs. Psy-Ed groups, this would be an interactive effect of mediation.

Dunning D, *et al. Evid Based Ment Health* 2022; 25:110–116. doi: 10.1136/ebmental-2022-300460

Table S1: Primary and secondary mental health and affective cognitive control outcomes across the main trial time points.

Measure	T/Q	T1	T2	T4
**Primary mental health outcomes**				
Depression (CES-D)	Q	✔	✔	✔
Social/emotional/behavioural functioning (SDQ)	Q	✔	✔	✔
Well-being (WEMWBS)	Q	✔	✔	✔
**Secondary mental health outcomes**				
Difficulties in Emotion regulation (DERS)	Q	✔	✔	✔
Anxiety (RCADS)	Q	✔	✔	✔
Ruminative Response Scale (RRS)	Q	✔	✔	✔
Mindfulness skills (CAMM)	Q	✔	✔	✔
**Affective executive control outcomes**				
Affective working memory	T	✔	✔	
Affective Stroop	T	✔	✔	
Affective sustained attention	T	✔	✔	
Executive processing (BRIEF-2) – self-report	Q	✔	✔	
Non-verbal IQ (CCFT)	Q	✔		

T1 = Baseline. T2= post-intervention. T4= mid-COVID lockdown follow-up (20-44 months after T2). T: Task. Q: Questionnaire. a=administered; CES-D: Center for Epidemiological Studies Depression Scale. SDQ: Strengths and Difficulties Questionnaire. WEMWBS: Warwick-Edinburgh Mental Well-Being Scale. RCADS: Revised Child Anxiety and Depression Scale. DERS: Difficulties with Emotion Regulation Scale. RRS: Ruminative Response Scale. CAMM: Child and Adolescent Mindfulness Measure. BRIEF-2: Behaviour Rating Inventory of Executive Function Version 2. CCFT: Cattell Culture Fair Test

Dunning D, *et al. Evid Based Ment Health* 2022; 25:110–116. doi: 10.1136/ebmental-2022-300460

Table S2: Secondary mental health outcomes at Baseline (T1)

	MT		Psy-Ed	
	**N**	**mean (SD)**	**N**	**mean (SD)**
SDQ-Emotion	219	3.8 (2.5)	209	3.9 (2.6)
SDQ-Conduct	219	2.1 (1.7)	208	2.3 (1.8)
SDQ-Hyperactivity	219	4.4 (2.4)	209	4.5 (2.5)
SDQ-Peer problems	219	2.1 (1.6)	209	2.4 (1.7)
SDQ-Prosocial	219	7.4 (1.9)	209	7.4 (1.9)
RCADS-Total	233	12.4 (7.7)	222	13.0 (8.0)
RCADS-Social	233	4.1 (2.2)	222	4.6 (2.4)
RCADS-Panic	231	1.7 (1.9)	221	1.8 (2.0)
RCADS-Separation	233	1.4 (1.8)	222	1.5 (1.7)
RCADS-Generalised	233	3.0 (2.1)	220	3.0 (2.1)
RCADS-Obsessive-Compulsive	233	1.9 (2.0)	220	2.0 (2.0)
RRS-Total	225	17.3 (6.0)	221	18.7 (6.9)

MT: Mindfulness Training. Psy-Ed: Psychoeducation training. SDQ: Strengths and Difficulties Questionnaire. SDQ subscales – Emotion: Emotional Symptoms; Conduct: Conduct Problems; Hyperactivity: Hyperactivity/Inattention; Peer problems: Peer Relationship Problems; Prosocial: Prosocial behaviour. RCADS: Revised Children’s Anxiety and Depression Scale. RCADS subscales – Social: social phobia; Panic: panic disorder Separation: separation anxiety disorder; Generalised: generalised anxiety disorder; Obsessive-Compulsive: obsessive compulsive disorder. RRS: Ruminative Response Scale.

Dunning D, *et al. Evid Based Ment Health* 2022; 25:110–116. doi: 10.1136/ebmental-2022-300460

Table S3: Additional affective executive control task variables at Baseline (Time 1)

	N	MT mean (SD)	N	Psy-Ed mean (SD)
SART-RT-NEG	193	350.3 (102.6)	193	355.8 (96.9)
SART-RT-NEU	193	347.6 (102.0)	192	352.1 (101.7)
SART-RT Contrast	193	2.7 (36.8)	192	4.0 (39.9)
SART-OM-NEG	193	17.2 (20.5)	191	18.3 (20.8)
SART-OM-NEU	193	16.9 (19.7)	190	16.3 (13.8)
SART-OM Contrast	193	0.3 (8.0)	190	1.5 (12.8)
SART-C-NEG	193	17.5 (6.0)	191	17.0 (6.5)
SART-C-NEU	193	17.1 (6.1)	191	16.7 (6.3)
SART-RTV-NEG	193	0.3 (0.1)	193	0.4 (0.2)
SART-RTV-NEU	193	0.3 (0.1)	192	0.3 (0.1)
STR-CON-RT	203	894.3 (243.1)	195	885.1 (235.5)
STR-NEU-RT	203	936.2 (270.1)	195	922.0 (260.6)
STR-INC-RT	203	957.6 (280.2)	195	946.9 (266.9)
STR-RT-INC minus NEU	203	21.4 (101.6)	195	24.9 (100.9)
STR-RT-CON minus NEU	203	41.9 (99.3)	195	36.8 (98.9)
STR-CON-AC	203	94.4 (7.5)	195	94.2 (8.2)
STR-NEU-AC	203	93.8 (7.1)	195	92.9 (8.7)
STR-INC-AC	203	90.8 (8.0)	195	90.1 (9.7)
WMC-NEG	226	0.7 (0.2)	216	0.7 (0.2)
WMC-NEU	223	0.7 (0.2)	208	0.7 (0.2)
WMC-ATDR	222	71.0 (15.0)	214	67.2 (17.7)
BRIEF-SB	228	8.3 (2.2)	215	8.4 (2.3)
BRIEF-SC	230	6.9 (1.6)	215	7.0 (1.5)
BRIEF-I	225	21.0 (5.1)	213	21.3 (5.2)
BRIEF-EC	226	17.2 (4.8)	212	18.1 (4.9)
BRIEF-M	230	8.0 (2.2)	216	8.0 (2.2)
BRIEF-WM	223	21.0 (4.6)	212	21.4 (4.8)
BRIEF-PO	227	22.3 (NA)	210	22.7 (4.5)
BRIEF-OoM	228	11.6 (2.9)	216	11.8 (3.1)
BRIEF-TC	229	17.2 (4.3)	214	17.5 (4.3)
DERS-Non-Acceptance	224	11.1 (4.7)	217	12.4 (5.7)
DERS-Goals	224	14.0 (4.7)	217	14.3 (5.2)
DERS-Impulse	224	12.2 (5.3)	217	12.9 (5.8)
DERS-Aware	231	19.3 (5.2)	222	18.1 (4.9)
DERS-Strategies	224	16.9 (7.2)	217	18.2 (8.0)

Dunning D, *et al. Evid Based Ment Health* 2022; 25:110–116. doi: 10.1136/ebmental-2022-300460

	N	MT<br>mean (SD)	N	Psy-Ed<br>mean (SD)
DERS-Clarity	231	11.8 (4.5)	222	12.3 (4.4)

MT: Mindfulness Training. Psy-Ed: Psychoeducation training. NEG: negative condition. NEU: neutral condition. RT: Reaction time. SART: Sustained Attention to Response Task. SART conditions – C: commission errors; OM: Omission errors; RTV: RT variance; Contrast: difference in performance between the negative condition and the neutral condition. STR: Stroop task. Stroop conditions – CON: congruent condition; INC: incongruent condition; AC: accuracy. WMC: Working Memory Capacity. ATDR: Attention target detection rate. BRIEF: Behaviour Rating Inventory of Executive Function version 2. BRIEF subscales – SB: Shift Behaviour; SC: Shift Cognitive, I: Inhibit, EC: Emotional Control M: Monitor, WM: Working Memory, P/O: Plan/Organize, OoM: Organization of Materials, TC: Task Completion. DERS: Difficulties with Emotion Regulation Scale.

Table S4: Zero-order Pearson correlations between key affective control variables and the primary mental health outcomes at trial Baseline (T1)

	**CES-D**	**WEMWBS**	**SDQ-Total**
DERS	.74***	-.65***	.72***
BRIEF	.48***	-.41***	.68***
CAMM	.48***	-.61***	.59***
aSTROOP	.02	-.001	-.007
STR-NEU-RT	.006	-.002	.04
aSART-C	.02	-.09	.03
aSART-RTV	-.13*	.11	-.13*
SART-C-NEU	.06	-.05	.08
SART-RTV-NEU	-.03	.02	.0001
aWMC	-.02	-.09	.01
WMC-NEU	.07	-.15**	-.09

CES-D: Center for Epidemiological Studies Depression Scale. SDQ-total: Strengths and Difficulties Questionnaire total score. WEMWBS: Warwick-Edinburgh Mental Well-Being Scale. DERS-Total: Difficulties with Emotion Regulation Scale total score. CAMM-total: Child and Adolescent Mindfulness Measure total score. BRIEF-Total: Behaviour Rating Inventory of Executive Function Version 2 Global composite score. aSART-C: Affective Sustained Attention to Response Task commission errors, computed as the number of errors in the negative condition minus the number in the neutral condition (SART-C-NEU). aSART-RTV: Affective Sustained Attention to Response Task reaction time variance, computed as RTV in negative condition minus the RTV in the neutral condition (SART-RTV-NEU). aStroop: Affective Stroop performance computed as mean reaction time in the incongruent trials minus mean reaction time in the congruent trials. aWMC: Affective Working Memory Capacity, computed as the proportion of correctly remembered words in the neutral condition (WMC-NEU) minus the proportion remembered in the negative condition. SART-C-NEU, SART-RTV-NEU, STR-NEU-RT (RT on the Stroop task for the neutral stimuli), and WMC-NEU represent the underlying task performance in the non-affective context. All 4 indices correlate also significantly with age, rs>|.15|, ps<.01, and verbal IQ on the Cattell Culture Fair Test at Baseline, rs>|.12|, ps<.01, with older age and higher IQ associated with better performance, as would be expected.

* p<.05; **p<.01, ***p<.001

Table S5: Differences between complete cases and those lost to follow up at post-intervention (Time 2)

	**N**	**Remaining mean (SD)**	**N**	**Lost to follow up mean (SD)**
Female	253	-	53	-
Male	147		7	
Age	400	13.7 (1.3)	60	14.7 (1.6)
London	272	-	56	-
Cambridge	128	-	4	-
IQ	394	111.3 (16.5)	55	101.3 (16.8)
SDQ-total	195	12.7 (5.7)	230	12.8 (5.6)
CES-D total	397	16.1 (10.2)	53	19.9 (9.4)
WEMWBS	397	47.5 (9.8)	54	45.1 (8.7)

IQ: score on the Cattell Culture Fair Test; CES-D: Center for Epidemiological Studies Depression Scale. SDQ-total: Strengths and Difficulties Questionnaire total score. WEMWBS: Warwick-Edinburgh Mental Well-Being Scale.

Dunning D, *et al. Evid Based Ment Health* 2022; 25:110–116. doi: 10.1136/ebmental-2022-300460

Table S6: Differences between complete cases and those lost to follow up at mid-pandemic (T4)

	**N**	**Remaining mean (SD)**	**N**	**Lost to follow up mean (SD)**
Female	135	-	171	-
Male	72		82	
Age	207	13.8 (1.3)	253	13.9 (1.4)
London	129	-	199	-
Cambridge	78	-	54	-
IQ	205	112.5 (15.8)	244	108.0 (17.4)
SDQ-total	195	12.7 (5.7)	230	12.8 (5.6)
CES-D total	206	17.1 (10.6)	244	16.1 (9.7)
WEMWBS	206	46.8 (9.6)	245	47.7 (9.7)

IQ: score on the Cattell Culture Fair Test; CES-D: Center for Epidemiological Studies Depression Scale. SDQ-total: Strengths and Difficulties Questionnaire total score. WEMWBS: Warwick-Edinburgh Mental Well-Being Scale.

Dunning D, *et al. Evid Based Ment Health* 2022; 25:110–116. doi: 10.1136/ebmental-2022-300460

Table S7: Moderating effects of gender and age on the relationship between intervention received and the primary mechanisms outcomes at post-intervention (T2)

**Moderation by gender**	**Interaction coefficient**	**95% CI**	**p-value**
CAMM-Total	-1.19	-3.39 to 1.01	.29
BRIEF-Total	-.46	-7.81 to 6.89	.90
DERS-Total	.24	-6.66 to 7.15	.95
aSART-C	.24	-1.64 to 2.12	.80
aSART-RTV	.002	-.05 to .05	.93
aSTROOP	8.29	-33.40 to 49.98	.70
aWMC	.03	-.05 to .11	.46
**Moderation by age**	**Interaction coefficient**	**95% CI**	**p-value**
CAMM-Total	.73	-.05 to 1.50	.07
BRIEF-Total	-1.57	-4.57 to 1.52	.30
DERS-Total	.70	-1.99 to 3.38	.61
aSART-C	.28	-.45 to 1.02	.45
aSART-RTV	.01	-.26 to .44	.61
aSTROOP	3.20	-12.67 to 19.08	.69
aWMC	-.01	-.05 to .03	.55

Inferential statistics are on the full imputed dataset (N=460). The study was not fully-powered for moderation as results are therefore exploratory. Coefficients quantify the moderating effects of gender or age on the relationship between the intervention received and the mechanism outcome. In our protocol, we also indicated that we would examine moderation by pubertal status but there were insufficient data completeness for these analyses due to the sensitivity of the question. Age, gender, intervention arm, location (London, Cambridge) and the Baseline (T1) score on the relevant outcome are included in the models. DERS-Total: Difficulties with Emotion Regulation Scale total score. CAMM-total: Child and Adolescent Mindfulness Measure total score. BRIEF-Total: Behaviour Rating Inventory of Executive Function version 2 Global Composite score. aSART-C: Affective Sustained Attention to Response Task commission errors, computed as the number of errors in the negative condition minus the number in the neutral condition. aSART-RTV: Affective Sustained Attention to Response Task reaction time variance, computed as RTV in negative condition minus the RTV in the neutral condition. aStroop: Affective Stroop performance computed as mean reaction time in the incongruent trials minus mean reaction time in the congruent trials. aWMC: Affective Working Memory Capacity, computed as the proportion of correctly remembered words in the neutral condition minus the proportion remembered in the negative condition.

Dunning D, *et al. Evid Based Ment Health* 2022; 25:110–116. doi: 10.1136/ebmental-2022-300460

Table S8: Additional cognitive task variables at post-intervention (T2)

	MT		Psy-Ed		Unadjusted (I – C)	Adjusted (I-C)		
	**N**	**mean (SD)**	**N**	**mean (SD)**	**mean diff.**	**mean diff.**	**95% CI**	**p-value**
SART-RT-NEG	166	357.5 (108.2)	157	354.4 (105.7)	2.8	6.7	-12.5 to 25.8	0.49
SART-RT-NEU	166	354.0 (107.9)	157	357.1 (104.5)	-4.9	-2.0	-20.6 to 16.7	0.84
SART-RT Contrast	166	3.5 (34.0)	157	-2.7 (32.3)	7.4	7.7	-0.2 to 15.6	0.06
SART-OM-NEG	168	19.4 (24.2)	157	17.0 (16.9)	3.6	3.9	-1.1 to 8.9	0.12
SART-OM-NEU	168	18.3 (21.7)	157	17.8 (16.4)	0.8	0.7	-3.8 to 5.1	0.77
SART-OM Contrast	168	1.2 (15.0)	157	-0.8 (10.3)	2.8	2.7	-0.5 to 5.9	0.10
SART-C-NEG	168	15.0 (6.5)	157	15.7 (6.6)	-0.6	-1.0	-2.2 to 0.3	0.13
SART-C-NEU	168	15.1 (6.2)	157	15.5 (6.5)	-0.3	-0.5	-1.8 to 0.8	0.47
SART-RTV-NEG	168	0.3 (0.1)	158	0.4 (0.2)	-0.02	-0.02	-0.1 to 0.0	0.61
SART-RTV-NEU	168	0.3 (0.1)	158	0.4 (0.1)	-0.02	-0.01	-0.1 to 0.1	0.69
STR-CON-RT	172	840.8 (204.1)	162	870.6 (222.1)	-9.9	-16.6	-52.7 to 19.5	0.37
STR-NEU-RT	172	869.7 (210.8)	162	907.5 (233.0)	-29.0	-40.9	-75.4 to -6.3	0.02
STR-INC-RT	172	878.9 (211.4)	162	924.9 (246.9)	-28.9	-38.4	-75.7 to -1.0	0.04
STR-RT-INC minus NEU	172	9.2 (74.2)	162	17.4 (93.1)	0.04	1.2	-17.6 to 19.9	0.90
STR-RT-CON minus NEU	172	28.9 (74.8)	162	36.9 (82.8)	-18.8	-20.5	-38.5 to -2.6	0.03
STR-CON-AC	172	94.1 (9.5)	162	94.4 (5.6)	-0.3	-0.4	-2.3 to 1.6	0.72
STR-NEU-AC	172	92.9 (9.4)	162	93.3 (6.3)	-0.5	-0.8	-2.8 to 1.2	0.45
STR-INC-AC	172	90.7 (9.6)	162	91.3 (6.2)	-0.9	-1.2	-3.0 to 0.7	0.23
WMC-NEG	182	0.8 (0.2)	180	0.8 (0.2)	-0.01	-0.02	-0.1 to 0.0	0.44
WMC-NEU	152	0.8 (0.2)	157	0.8 (0.2)	-0.03	-0.03	-0.1 to 0.0	0.35
WMC-ATDR	149	72.4 (15.2)	159	69.0 (18.1)	3.4	1.4	-2.8 to 5.6	0.52

	MT		Psy-Ed		Unadjusted (I – C)	Adjusted (I-C)		
	**N**	**mean (SD)**	**N**	**mean (SD)**	**mean diff.**	**mean diff.**	**95% CI**	**p-value**
BRIEF-SB	201	8.1 (2.2)	186	8.5 (2.5)	-0.4	-0.4	-0.8 to 0.1	0.09
BRIEF-SC	203	6.8 (1.5)	185	6.9 (1.6)	-0.1	-0.1	-0.4 to 0.2	0.64
BRIEF-I	198	21.0 (5.3)	187	21.3 (5.5)	-0.3	-0.1	-1.0 to 0.8	0.87
BRIEF-EC	202	17.2 (4.9)	184	18.0 (5.3)	-0.9	-0.3	-1.1 to 0.6	0.51
BRIEF-M	202	7.9 (2.2)	187	8.1 (2.4)	-0.04	-0.05	-0.5 to 0.4	0.83
BRIEF-WM	199	21.3 (5.1)	185	21.6 (5.1)	-0.7	-0.4	-1.3 to 0.5	0.38
BRIEF-PO	200	22.5 (4.7)	185	23.0 (4.8)	-0.9	-0.6	-1.6 to 0.3	0.19
BRIEF-OoM	199	11.7 (3.0)	185	12.2 (3.2)	-0.6	-0.4	-0.9 to 0.1	0.10
BRIEF-TC	198	17.2 (4.2)	185	17.4 (4.3)	-0.5	-0.3	-1.1 to 0.4	0.39
DERS-Non-acceptance	201	11.3 (4.7)	194	11.9 (5.8)	0.3	0.4	-0.7 to 1.5	0.50
DERS-Goals	201	14.1 (5.1)	195	13.8 (5.2)	0.6	0.6	-0.5 to 1.6	0.29
DERS-Impulse	202	12.5 (5.2)	195	13.1 (5.7)	0.1	0.2	-0.9 to 1.2	0.77
DERS-Awareness	202	18.5 (5.5)	195	18.3 (5.1)	-0.7	-0.6	-1.8 to 0.5	0.29
DERS-Strategies	201	17.3 (7.1)	195	18.2 (7.8)	-0.1	-0.06	-1.6 to 1.5	0.94
DERS-Clarity	202	12.1 (4.1)	195	11.8 (4.2)	0.5	0.5	-0.4 to 1.3	0.26

Data presented are complete cases. Inferential statistics are on the full imputed dataset (N=460). MT: Mindfulness Training. Psy-Ed: Psychoeducation training. I: Intervention (MT). C: Control (Psy-Ed). NEG: negative condition. NEU: neutral condition. RT: Reaction time. SART: Sustained Attention to Response Task. SART conditions –; C: commission errors; OM: Omission errors; RTV: RT variance; Contrast: difference in performance between the negative condition and the neutral condition. STR: Stroop task. Stroop conditions – CON: congruent condition; INC: incongruent condition; AC: accuracy. WMC: Working Memory Capacity. ATDR: Attention target detection rate. BRIEF: Behaviour Rating Inventory of Executive Function version 2. BRIEF subscales – SB: Shift Behaviour; SC: Shift Cognitive, I: Inhibit, EC: Emotional Control M: Monitor, WM: Working Memory, P/O: Plan/Organize, OoM: Organization of Materials, TC: Task Completion. DERS: Difficulties with Emotion Regulation Scale.

Table S9: Zero-order Pearson correlations between pre- to post-intervention change (Δ) in primary affective control variables and in mental health outcomes

	**ΔCES-D**	**ΔWEMWBS**	**ΔSDQ-Total**
ΔDERS	.15*	-.15*	.10
ΔBRIEF	.33***	-.21***	.42***
ΔCAMM	.04	-.005	.04
ΔaSTROOP	.05	-.04	-.04
ΔSTR-NEU-RT	.07	-.04	.05
ΔaSART-C	.04	-.11	-.008
ΔaSART-RTV	-.14*	.06	-.14*
ΔSART-C-NEU	.06	-.05	.10
ΔSART-RTV-NEU	.05	-.04	.05
ΔaWMC	-.02	-.09	.01
ΔWMC-NEU	.02	-.002	.03

CES-D: Center for Epidemiological Studies Depression Scale. SDQ-total: Strengths and Difficulties Questionnaire total score. WEMWBS: Warwick-Edinburgh Mental Well-Being Scale. DERS-Total: Difficulties with Emotion Regulation Scale total score. CAMM-total: Child and Adolescent Mindfulness Measure total score. BRIEF-Total: Behaviour Rating Inventory of Executive Function Version 2 Global composite score. aSART-C: Affective Sustained Attention to Response Task commission errors, computed as the number of errors in the negative condition minus the number in the neutral condition (SART-C-NEU). aSART-RTV: Affective Sustained Attention to Response Task reaction time variance, computed as RTV in negative condition minus the RTV in the neutral condition (SART-RTV-NEU). aStroop: Affective Stroop performance computed as mean reaction time in the incongruent trials minus mean reaction time in the congruent trials. aWMC: Affective Working Memory Capacity, computed as the proportion of correctly remembered words in the neutral condition (WMC-NEU) minus the proportion remembered in the negative condition. SART-C-NEU, SART-RTV-NEU, STR-NEU-RT (RT on the Stroop task for the neutral stimuli), and WMC-NEU represent the underlying task performance in the non-affective context.

* p<.05; ***p<.001

Table S10: Secondary mental health outcomes at the mid-pandemic primary endpoint (T3) and at post-intervention (T2)

		MT		Psy-Ed		Unadjusted (I – C)	Adjusted (I-C)		
	**Timepoint**	**N**	**mean (SD)**	**N**	**mean (SD)**	**mean diff.**	**mean diff.**	**95% CI**	**p-value**
SDQ-Emotion	post-intervention	204	3.5 (2.5)	196	4.0 (2.6)	-0.5	-0.5	-1.0 to 0.0	0.06
	mid-pandemic	104	4.1 (2.3)	101	4.1 (2.9)	0.1	0.1	-0.5 to 0.8	0.69
SDQ-Conduct	post-intervention	204	2.0 (1.6)	196	2.2 (1.7)	-0.04	-0.04	-0.4 to 0.3	0.82
	mid-pandemic	104	2.4 (1.5)	101	1.9 (1.7)	0.6	0.6	0.1 to 1.1	0.01
SDQ-Hyperactivity	post-intervention	203	4.3 (2.4)	196	4.4 (2.4)	-0.2	-0.2	-0.7 to 0.2	0.34
	mid-pandemic	104	4.6 (2.2)	101	4.5 (2.3)	0.3	0.3	-0.3 to 0.9	0.28
SDQ-Peer problems	post-intervention	204	2.1 (1.6)	196	2.3 (1.9)	-0.2	-0.2	-0.5 to 0.2	0.31
	mid-pandemic	104	2.2 (1.6)	101	2.7 (1.9)	-0.3	-0.3	-0.9 to 0.2	0.21
SDQ-Prosocial	post-intervention	204	7.3 (1.9)	196	7.3 (1.9)	-0.1	-0.1	-0.5 to 0.3	0.60
	mid-pandemic	104	7.7 (2.0)	101	7.3 (2.0)	0.4	0.4	-0.1 to 0.9	0.15
RCADS-Total	post-intervention	203	11.9 (7.3)	196	12.9 (8.7)	-0.2	-0.2	-1.5 to 1.2	0.82
	mid-pandemic	104	12.4 (7.3)	100	12.3 (8.3)	1.3	1.3	-0.3 to 3.0	0.12
RCADS-Social	post-intervention	202	4.2 (2.2)	195	4.6 (2.4)	0.06	0.07	-0.4 to 0.5	0.76
	mid-pandemic	104	4.9 (2.2)	100	4.9 (2.6)	0.4	0.5	-0.2 to 1.1	0.14
RCADS-Panic	post-intervention	201	1.6 (1.9)	195	1.8 (2.2)	-0.1	-0.1	-0.5 to 0.3	0.55
	mid-pandemic	104	1.8 (2.0)	100	1.9 (2.1)	0.03	0.04	-0.5 to 0.5	0.89
RCADS-Separation	post-intervention	202	1.3 (1.6)	196	1.6 (1.8)	-0.05	-0.05	-0.4 to 0.3	0.76
	mid-pandemic	104	1.4 (1.6)	100	1.2 (1.6)	0.3	0.3	-0.2 to 0.7	0.23
RCADS-Generalised	post-intervention	201	2.8 (2.1)	195	2.9 (2.2)	-0.1	-0.1	-0.5 to 0.3	0.51
	mid-pandemic	104	2.6 (1.9)	100	2.7 (2.1)	0.1	0.1	-0.4 to 0.6	0.60
	post-intervention	202	1.8 (2.0)	194	1.9 (2.2)	0.04	0.04	-0.3 to 0.4	0.86

			MT		Psy-Ed	Unadjusted (I – C)	Adjusted (I-C)		
	**Timepoint**	**N**	**mean (SD)**	**N**	**mean (SD)**	**mean diff.**	**mean diff.**	**95% CI**	**p-value**
RCADS-Obsessive-Compulsive	mid-pandemic	104	1.4 (1.9)	100	1.3 (1.8)	0.1	0.1	-0.4 to 0.6	0.57
RRS-Total	post-intervention	203	17.7 (5.8)	195	19.1 (6.9)	-0.6	-0.6	-1.9 to 0.7	0.38
	mid-pandemic	104	17.9 (6.0)	100	18.1 (6.4)	-1.4	-1.4	-3.2 to 0.4	0.13

Data presented are complete cases. Inferential statistics are on the full imputed dataset (N=460). MT: Mindfulness Training. Psy-Ed: Psychoeducation training. I: Intervention (MT). C: Control (Psy-Ed). SDQ: Strengths and difficulties Questionnaire. SDQ subscales – Emotion: Emotional Symptoms; Conduct: Conduct Problems; Hyperactivity: Hyperactivity/Inattention; Peer problems: Peer Relationship Problems; Prosocial: Prosocial behaviour. RCADS: Revised Children's Anxiety and Depression Scale. RCADS subscales – Social: social phobia; Panic: panic disorder Separation: separation anxiety disorder; Generalised: generalised anxiety disorder; Obsessive-Compulsive: obsessive compulsive disorder.

Table S11: Primary and secondary mental health outcomes at the additional 3-month follow up assessment

	MT		Psy-Ed		Unadjusted (I-C)	Adjusted (I-C)		
	**N**	**mean (SD)**	**N**	**mean (SD)**	**mean diff.**	**mean diff.**	**95% CI**	**p-value**
CES-D	131	14.4 (10.0)	110	14.8 (10.0)	-0.0009	0.05	-2.3 to 2.4	0.97
SDQ-Total	131	10.8 (5.8)	109	11.4 (5.6)	0.6	0.6	-0.8 to 2.1	0.40
SDQ-Emotion	131	3.3 (2.5)	109	3.7 (2.4)	-0.2	-0.2	-0.9 to 0.5	0.55
SDQ-Conduct	131	1.7 (1.6)	109	1.8 (1.7)	0.4	0.4	-0.1 to 0.9	0.13
SDQ-Hyperactivity	131	3.9 (2.4)	109	3.8 (2.4)	0.3	0.3	-0.4 to 0.9	0.39
SDQ-Peer problems	131	1.8 (1.7)	109	2.2 (1.7)	0.06	0.06	-0.4 to 0.5	0.78
SDQ-Prosocial	131	7.6 (1.8)	109	7.7 (1.9)	-0.6	-0.6	-1.1 to -0.1	0.02
WEMWBS	131	49.2 (9.5)	109	49.5 (9.4)	-0.9	-1.0	-3.2 to 1.3	0.41
RCADS-Total	131	11.3 (7.8)	109	12.6 (9.1)	-1.2	-1.2	-2.9 to 0.5	0.18
RCADS-Social	131	4.0 (2.2)	109	4.3 (2.4)	0.2	0.2	-0.3 to 0.7	0.44
RCADS-Panic	130	1.5 (2.0)	109	1.7 (2.2)	-0.4	-0.4	-1.0 to 0.1	0.13
RCADS-Separation	131	1.4 (1.7)	109	1.8 (2.1)	-0.4	-0.4	-0.8 to 0.0	0.04
RCADS-Generalised	131	2.6 (2.1)	109	2.8 (2.1)	-0.2	-0.2	-0.8 to 0.3	0.36
RCADS-Obsessive Compulsive	131	1.6 (1.9)	109	1.6 (2.2)	-0.3	-0.3	-0.8 to 0.2	0.31
RRS-total	131	16.5 (6.4)	107	18.1 (7.0)	0.3	0.3	-1.5 to 2.2	0.73

Data presented are complete cases. Inferential statistics are on the full imputed dataset (N=460). MT: Mindfulness Training. Psy-Ed: Psychoeducation training. I: Intervention (MT). C: Control (Psy-Ed). CES-D: Center for Epidemiologic Studies for Depression Scale. SDQ-total: Strengths and Difficulties Questionnaire total score. WEMWBS: Warwick-Edinburgh Mental Well-Being Scale.
SDQ subscales – Emotion: Emotional Symptoms; Conduct: Conduct Problems; Hyperactivity: Hyperactivity/Inattention; Peer problems: Peer Relationship Problems; Prosocial: Prosocial behaviour. RCADS: Revised Children's Anxiety and Depression Scale. RCADS subscales – Social: social phobia; Panic: panic disorder Separation: separation anxiety disorder; Generalised: generalised anxiety disorder; Obsessive-Compulsive: obsessive compulsive disorder.

Supplementary references

1. Schweizer S, Dalgleish T. The impact of affective contexts on working memory capacity in healthy populations and in individuals with PTSD. *Emotion.* 2016;16(1):16.
2. Robertson IH, Manly T, Andrade J, et al. Oops!': performance correlates of everyday attentional failures in traumatic brain injured and normal subjects. *Neuropsychologia*. 1997;35(6):747-58.
3. Bradley MM, Lang PJ. Affective reactions to acoustic stimuli. *Psychophysiology.* 2000;37(2):204-15.
4. Preston SD, Stansfield RB. I know how you feel: Task-irrelevant facial expressions are spontaneously processed at a semantic level. *Cognitive, Affective, & Behavioral Neuroscience.* 2008;8(1):54-64.
5. Egger HL, Pine DS, Nelson E, et al. The NIMH Child Emotional Faces Picture Set (NIMH-ChEFS): a new set of children's facial emotion stimuli. *Int J Methods Psychiatr Res*. 2011;20(3):145-56.
6. Thomas KM, Drevets WC, Whalen PJ, et al. Amygdala response to facial expressions in children and adults. *Biol Psychiatry*. 2001;49(4):309-16.
7. Kuperman V, Stadthagen-Gonzalez H, Brysbaert M. Age-of-acquisition ratings for 30,000 English words. *Behav Res Methods*. 2012;44(4):978-90.
8. Cattell RB. Theory of fluid and crystallized intelligence: A critical experiment. *J Educ Psychol*. 1963;54(1):1.
9. Radloff LS. The use of the Center for Epidemiologic Studies Depression Scale in adolescents and young adults. *J Youth Adolesc.* 1991;20(2):149-66.
10. Goodman R. The Strengths and Difficulties Questionnaire: a research note. *J Child Psychol Psychiatry.* 1997;38(5):581-6.
11. Tennant R, Hiller L, Fishwick R, et al. The Warwick-Edinburgh mental well-being scale (WEMWBS): development and UK validation. *Health Qual Life Outcomes.* 2007;5(1):1-3.
12. Gioia G. A, Isquith P. K, Guy, S. C et al. Behavior Rating Inventory of Executive Function®, Second Edition (BRIEF®2). Lutz, FL: PAR Inc.2015.
13. Greco LA, Baer RA, Smith GT. Assessing mindfulness in children and adolescents: development and validation of the Child and Adolescent Mindfulness Measure (CAMM). *Psychol Assess.* 2011;23(3):606.
14. Chorpita BF, Yim L, Moffitt C, et al. Assessment of symptoms of DSM-IV anxiety and depression in children: A revised child anxiety and depression scale. *Behav Res Ther.* 2000;38(8):835-55.
15. Gratz KL, Roemer L. Multidimensional assessment of emotion regulation and dysregulation: Development, factor structure, and initial validation of the difficulties in emotion regulation scale. *J Psychopathol Behav Assess.* 2004;26(1):41-54.
16. Nolen-Hoeksema S. The role of rumination in depressive disorders and mixed anxiety/depressive symptoms. *J Abnorm Psychol.* 2000;109(3):504.
17. Treynor, W., Gonzalez, R., Nolen-Hoeksema, S. Rumination reconsidered: A psychometric analysis. *Cog. Ther. Res.*, 2003;27(3): 247-259.
18. Grund S, Lüdtke O, Robitzsch A. Multiple imputation of multilevel missing data: An introduction to the R package pan. *Sage Open*. 2016;6(4):2158244016668220.
19. Kraemer HC, Wilson GT, Fairburn CG, et al. Mediators and moderators of treatment effects in randomized clinical trials. *Arch Gen Psychiatry*. 2002;59(10):877-83.
